# Clinical Antibiotic-resistant *Acinetobacter baumannii* Strains with Higher Susceptibility to Environmental Phages than Antibiotic-sensitive Strains

**DOI:** 10.1038/s41598-017-06688-w

**Published:** 2017-07-24

**Authors:** Li-Kuang Chen, Shu-Chen Kuo, Kai-Chih Chang, Chieh-Chen Cheng, Pei-Ying Yu, Chih-Hui Chang, Tren-Yi Chen, Chun-Chieh Tseng

**Affiliations:** 1 Institute of Medical Sciences, Department of Laboratory Diagnostic, College of Medicine, Tzu Chi University, Hualien, Taiwan; 2 Department of Laboratory Medicine, Clinical Pathology, Buddhist Tzu Chi General Hospital, Hualien, Taiwan; 3 National Institute of Infectious Diseases and Vaccinology, National Health Research Institutes, Miaoli County, Taiwan; 4 Department of Laboratory Medicine and Biotechnology, Tzu Chi University, Hualien, Taiwan; 5 Department and Graduate Institute of Public Health, Tzu Chi University, Hualien, Taiwan; 6 Emergency Department, Changhua Christian Hospital, Changhua, Taiwan

## Abstract

Antibiotic-resistant *Acinetobacter baumannii* is associated with nosocomial infections worldwide. Here, we used clinically isolated *A. baumannii* strains as models to demonstrate whether antibiotic resistance is correlated with an increased susceptibility to bacteriophages. In this study, 24 active phages capable of infecting *A. baumannii* were isolated from various environments, and the susceptibilities of both antibiotic-sensitive and antibiotic-resistant strains of *A. baumannii* to different phages were compared. In our study, a total of 403 clinically isolated *A. baumannii* strains were identified. On average, the phage infection percentage of the antibiotic-resistant *A. baumannii* strains was 84% (from 81–86%), whereas the infection percentage in the antibiotic-sensitive *A. baumannii* strains was only 56.5% (from 49–64%). In addition, the risk of phage infection for *A. baumannii* was significantly increased in the strains that were resistant to at least four antibiotics and exhibited a dose-dependent response (*p*-trend < 0.0001). Among all of the *A. baumannii* isolates, 75.6% were phage typeable. The results of phage typing might also reveal the antibiotic-resistant profiles of clinical *A. baumannii* strains. In conclusion, phage susceptibility represents an evolutionary trade-off in *A. baumannii* strains that show adaptations for antibiotic resistance, particularly in medical environments that have high antibiotic use.

## Introduction

*Acinetobacter baumannii* is a gram-negative coccobacillus that has been listed as the most important pathogen in hospitals^1^. *A. baumannii* is an opportunistic pathogen with a wide spectrum, and it has been described as a dangerous pathogen by the Infectious Disease Society of America^2^. Following the introduction of antibiotics into clinical use, *A. baumannii* has become problematic in intensive care units (ICUs) because it has developed resistance to broad-spectrum antibiotics^3^, and the incidence of multidrug-resistant *A. baumannii* (MDRAB) infections has continued to increase worldwide^4^.

Because of the rapid accumulation of resistance to multiple antibiotics, carbapenems are the only effective group of antibiotics against MDRAB infections. Carbapenems are classified as a subgroup of the β-lactam antibiotics. Similar to other β-lactams, carbapenems can bind to the penicillin-binding proteins of bacteria, which inhibits cell wall synthesis and results in cell death^1^. Unfortunately, the number of *A. baumannii* strains showing resistance to carbapenems has been increasing rapidly^5^. Although last resort treatments are available for MDRAB, such as colistin^6^, these antibiotics may cause severe physical side effects, including neurotoxicity or nephrotoxicity^6^.

Bacteriophage (phage) therapy represents a potential alternative strategy for treating infections with multidrug-resistant (MDR) strains. Phages are host-specific, natural parasites of bacteria. Because antibiotic-resistant bacteria are a serious health concern, the use of phages to reduce the concentration of specific bacterial pathogens has experienced renewed interest^7^. The first active phage shown to specifically infect MDRAB was characterized in 2010^8^, and several isolated phage strains capable of infecting MDRAB have subsequently been identified^9–11^. Our group also isolated *A. baumannii*-specific phages for different applications^8, 12–15^.

Twenty-four new phage strains have been isolated by our group, and we expect that these phages have the potential for use as biocontrol agents^16^ and could be applied in clinical and environmental settings^15^. Before applying these phages for therapy or environmental cleaning, the susceptibility of clinically isolated *A. baumannii* strains to these 24 phages should be determined. The development of resistance to different antibiotics may be correlated with the evolution of resistance to phages^17^. This antagonistic coevolutionary relationship among bacteria, antibiotics and phages must be investigated via studies focused on clinical and environmental settings. Although *in vitro* tests have been conducted to explore the cross-resistance profile to antibiotics and phages using a single bacterial strain^17–19^, the cross-resistance profiles of clinical bacteria to multiple antibiotics and environmental phages has not been studied.

Here, we hypothesize that the use of antibiotics in clinical settings may affect the evolution of bacterial resistance to phages. Consequently, we used clinically isolated *A. baumannii* strains as models to demonstrate the correlation between bacterial resistance to antibiotics and phages. The susceptibilities of both antibiotic-sensitive and antibiotic-resistant *A. baumannii* to 24 phage strains were compared. Using this approach, we determined how bacterial resistance to different types and numbers of antibiotics affects bacterial susceptibility to phages. This study was conducted to determine whether the development of resistance to different types and numbers of antibiotics in *A. baumannii* generated significant fitness costs that impacted the evolution of resistance to phages in *A. baumannii*.

## Results

### Drug-resistant profiles of the clinically isolated *A. baumannii*

**Figure 1:**
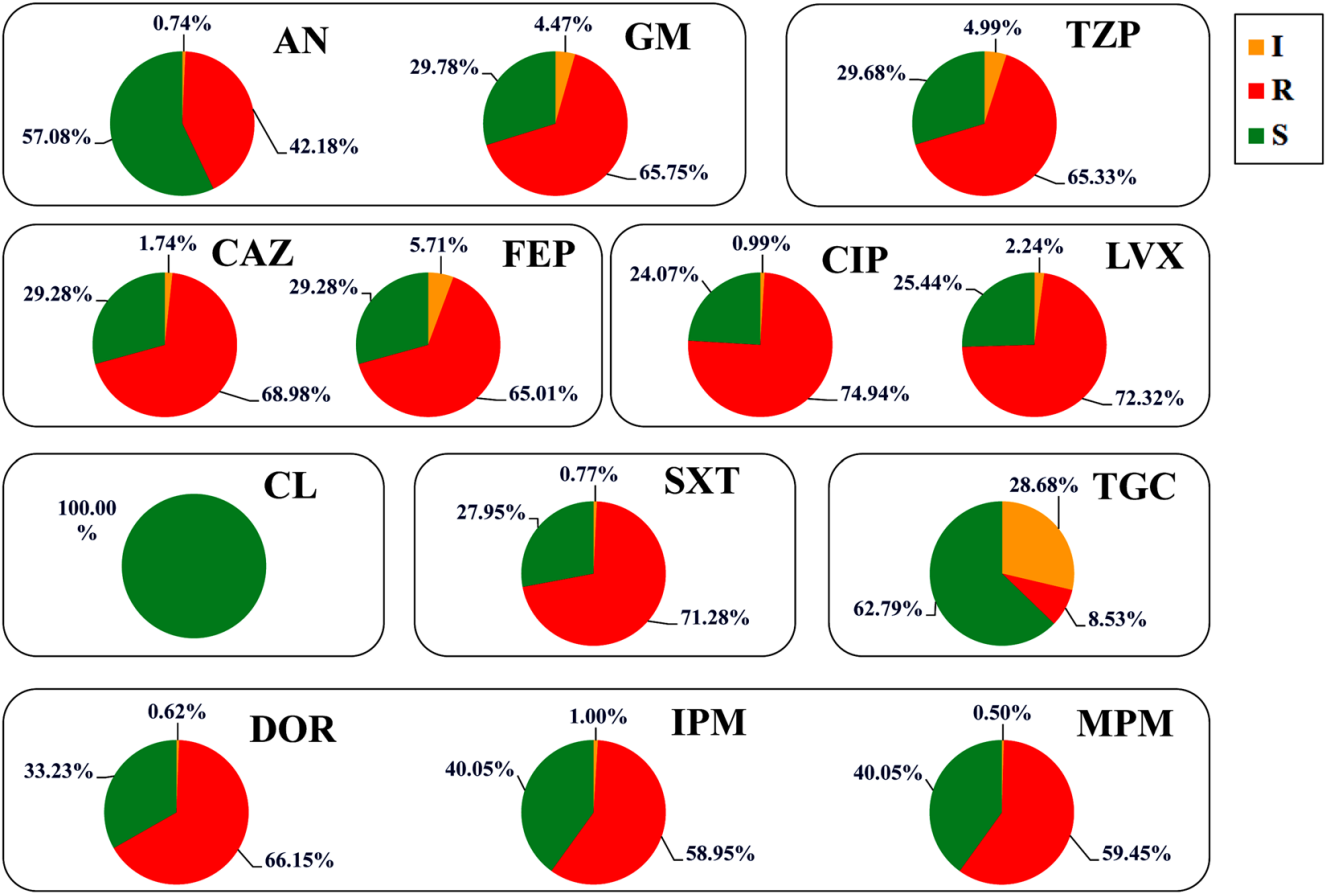
*In vitro* antibiotic susceptibilities of the clinical isolates of *A. baumannii* determined using the broth-dilution method: S (susceptible), R (resistance), and I (intermediate). The antibiotics in this study were divided into eight categories: (I) Aminoglycoside (amikacin and gentamicin), (II) Beta-lactam/beta-lactamase inhibitor combination (tazobactam-piperacillin), (III) Cephalosporin (ceftazidime and cefepime), (IV) Quinolone (ciprofloxacin and levofloxacin), (V) Polymyxin (colistin), (VI) Sulfonamides (baktar), (VII) Tetracycline (tigecycline) and (VIII) Carbapenem (doripenem, imipenem and meropenem).

In this study, we divided the clinically used antibiotics into eight categories according to their resistance mechanisms (Fig. 1). Most of the clinically isolated *A. baumannii* (>70%) were resistant to group IV (73.6%) and group VI (71.3%). In addition, more than half (from 50–70%) of the strains were resistant to group III (67%), group II (65.3%), group VIII (61.5%) and group I (54%). *A. baumannii* was more sensitive to the tigecycline and colistin groups compared with the six other groups. All of the *A. baumannii* strains were sensitive to colistin (group V; 0%), and only a small fraction was resistant to tigecycline (group VII; 8.5%). However, nearly 26.7% of the strains demonstrated intermediate (I) resistance to the glycylcycline antibiotic tigecycline.

### Association between resistance to phages and antibiotics

We observed a strong association between resistance to phages and antibiotics (except tigecycline) (Table 1). In addition, because the *A. baumannii* strains were all sensitive to colistin, the association between phage and antibiotic resistance was not available for group V. The *A. baumannii* strains showing resistance to six categories of antibiotics (excluding colistin and tigecycline) demonstrated higher phage infection percentages (IP%) than the strains showing antibiotic sensitivity. On average, the phage IP% of the antibiotic-resistant *A. baumannii* strains was 84% (from 81–86%), whereas the IP% of the antibiotic-sensitive *A. baumannii* was 56.5% (from 49–64%).

**Table 1:** Differences in the distributions of phage infection rates between *A. baumannii* strains showing resistance and sensitivity to the different antibiotic categories.

Antibiotic categories	Infected by phages	Resistant (%)	Sensitive (%)	*p*
I (N = 286)	Yes	136 (81)	72 (61)	0.0002^a^
	No	32 (19)	46 (39)	
II (N = 401)	Yes	219 (84)	84 (60)	<0.0001^a^
	No	43 (16)	55 (40)	
III (N = 364)	Yes	214 (84)	60 (55)	<0.0001^a^
	No	41 (16)	49 (45)	
IV (N = 387)	Yes	246 (85)	49 (50)	<0.0001^a^
	No	44 (15)	48 (50)	
V (N = 256)	Yes	N/A	213 (84)	N/A
	No	N/A	43 (16)	
VI (N = 390)	Yes	239 (86)	55 (49)	<0.0001^a^
	No	39 (14)	57 (51)	
VII (N = 258)	Yes	19 (91)	196 (83)	0.18^b^
	No	2 (9)	41 (17)	
VIII (N = 403)	Yes	200 (84)	105 (64)	<0.0001^a^
	No	39 (16)	59 (36)	

Notes: ^a^Chi-squared test. ^b^Fisher’s exact test. N/A = not available. Resistant = resistant to the respective antibiotic category. Sensitive = sensitive to the respective category. The antibiotic categories are as follows: (I) Aminoglycoside (amikacin and gentamicin), (II)B eta-lactam/beta-lactamase inhibitor combination (tazobactam-piperacillin), (III) Cephalosporin (ceftazidime and cefepime), (IV) Quinolone (ciprofloxacin and levofloxacin), (V) Polymyxin (colistin), (VI) Sulfonamides (baktar), (VII) Tetracycline (tigecycline) and (VIII) Carbapenem (doripenem, imipenem and meropenem).

### Correlation between phage infection risk and antibiotic resistance in *A. baumannii*

The results presented in Table 2 show that most *A. baumannii* strains isolated from clinical settings were antibiotic resistant, and a smaller proportion were antibiotic sensitive. Table 2 also indicates that in *A. baumannii*, the risk of infection by phages significantly increased in the strains that showed resistance to more antibiotics, and a dose-dependent response was observed (*p*-trend < 0.0001). The risk of phage infection in the strains resistant to 1–3 different antibiotics was 1.4 times higher than that of the sensitive strains; however, this difference was not significant (*p* = 0.462). The risk of phage infection among the *A. baumannii* strains resistant to more than 3 different antibiotics was 4 times higher than that of the sensitive strains (*p* = 0.003).

**Table 2:** Odds ratios (OR) and 95% confidence intervals (CIs) of the phage infection ratio according to the number of antibiotics to which the clinically isolated *A. baumannii* were resistant.

	Total (N)	Phage susceptibility pattern			95% CI	*p*
		Infected cases		OR		
		N	(%)			
Number of antibiotics						
0	79	39	49.3	1.00		
1–3	26	15	57.7	1.39	0.6–3.4	0.462
4–6	32	26	81.3	4.44	1.6–12.0	0.003
7–9	95	81	85.2	5.93	2.9–12.1	<0.0001
10–12	171	144	84.2	5.47	3.0–10.0	<0.0001
*p*-trend						<0.0001

OR and 95% CI values were estimated using binary logistic regression model.

The *p*-trend was calculated using the continuous scale of the number of resistant antibiotics in the corresponding models.

### Association between the profiles of phage infection and antibiotic resistance

**Figure 2:**
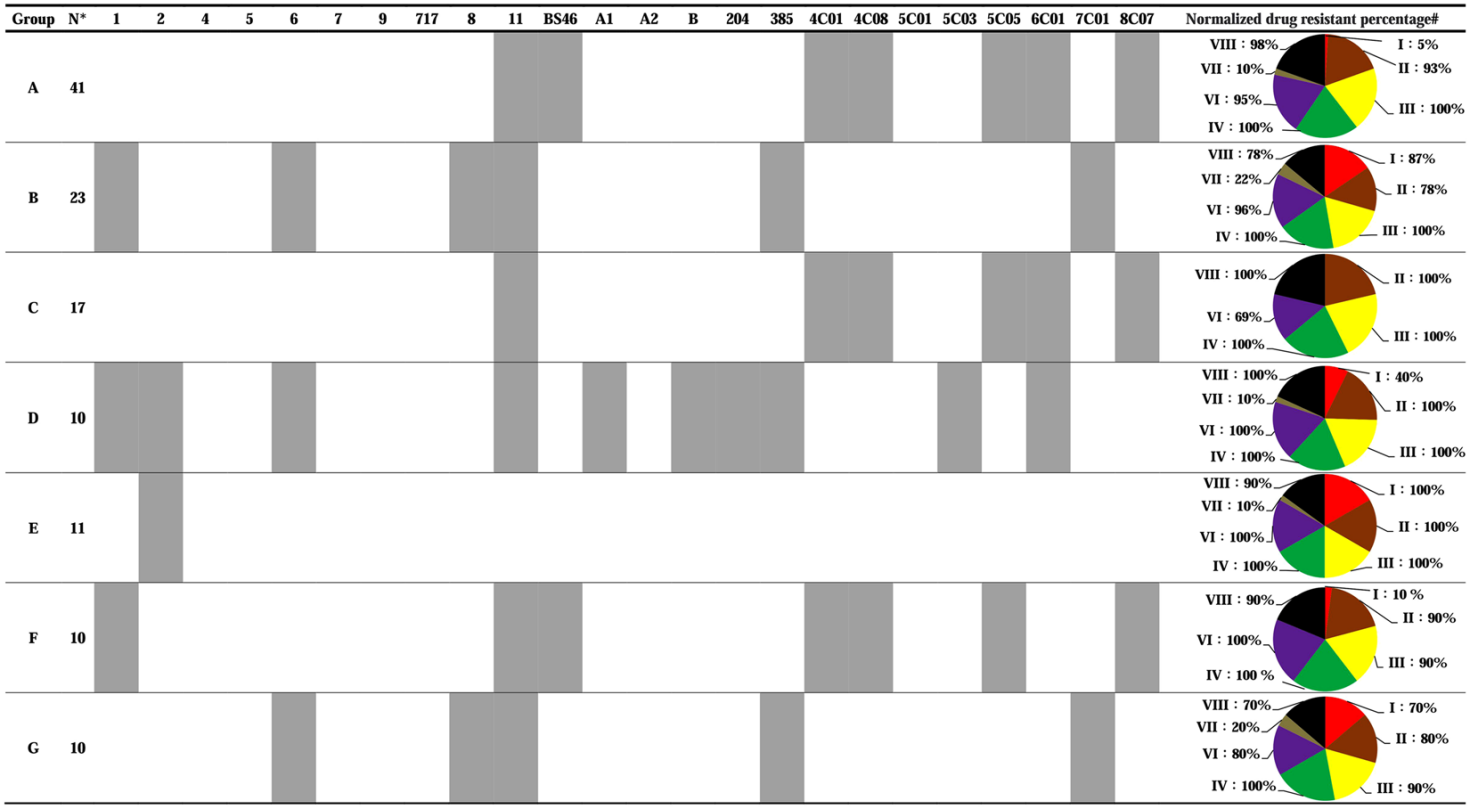
Phage infection profiles that contained at least 10 samples of clinically isolated *A. baumannii* (total N = 122), and the normalized drug resistance percentages to the eight groups of antibiotics. The different colors in the pie charts represent the different groups of antibiotics: Group I  (AN + GM), Group II  (TZP), Group III  (CAZ + FEP), Group IV  (CIP + LVX), Group V  (CL), Group VI  (SXT), Group VII  (TGC), and Group VIII  (DOR + IPM + MPM). The gray box indicates that *A. baumannii* can be infected by the indicated phage. *N = The number of clinically isolated *A. baumannii* represent the respective phage infection profiles. ^#^Normalized drug resistance percentage = the isolates of *A. baumannii* that are resistant to the respective group of antibiotics divided by the total isolates of *A. baumannii* (N) in each phage profile group and multiplied by 100.

From Fig. 2, we selected the top seven phage infection profiles and determined their corresponding resistance percentages to the eight groups of antibiotics. The top seven phage infection profiles that contained at least 10 samples from clinically isolated *A. baumannii* were selected. The remaining phage infection profiles that contained less than 10 samples are not shown here. For the seven selected phage infection profiles (group A to G), limited differences were observed among certain profiles. For example, the *A. baumannii* strains in groups A, C, and F and groups B and G could be infected by similar phages. However, groups D and E presented totally different infection profiles from the other groups. After combining the phage infection profiles with the antibiotic resistance information, a chi-squared test was performed, and the results indicated that five groups had similar infection profiles, and they also showed similar antibiotic resistance profiles. These 5 major groups included “group A and F (*p* = 0.056)”, “group B and G (*p* = 0.924)”, and groups C, D and E. Among these five different groups, considerable variations in the percentage distribution into the categories Aminoglycoside (group I; coefficient of variation (CV) = 79.8%) and Tetracycline (group VII; CV = 96.7%) were observed because these two antibiotic categories are effective for the treatment of *A. baumannii*.

## Discussion

In Taiwan, the first MDRAB strain that was resistant to almost all available antibiotics was discovered in 1998^20^. Subsequently, outbreaks caused by MDRAB have been frequently reported^21^. From 2007–2012, *A. baumannii* was the top pathogen responsible for nosocomial infections in ICUs, although its ranking has gradually declined over the past three years in Taiwan (from fifth to sixth)^22^. A Taiwan surveillance report in 2014 demonstrated that the percentage of antibiotic-resistant strains of clinically isolated *A. baumannii* was ordered as follows: group II (66%) > group III (62.5%) = group IV (62.5%) > group VIII (61%) > group I (59.5) > group VII (23%) > group V (1%)^22^. This order, which was reported in a previous study, was based on data collected from 21 medical centers, and it is similar to the results presented in the current study: group IV (73.6%) > group III (67%) > group II (65.3%) > group VIII (61.5%) > group I (54%) > group VII (8.5%) > group V (0%). Over 60% of clinical *A. baumannii* strains in Taiwan were resistant to quinolone, beta-lactam/beta-lactamase inhibitor, cephalosporin and carbapenem.

Antibiotic-resistant *A. baumannii* strains are prevalent in Taiwan and represent one of the most significant health-related problems in many areas of the world. A ten-year study in Spain (from 2004–2014) demonstrated that the percentage of antibiotic-resistant strains of *A. baumannii* to groups I (40%), IV (73.6%) and VIII (58.4%) was similar to the percentage observed in Taiwan^23^. Studies conducted in Saudi Arabia and Greece in 2014 indicated extremely high percentages of antibiotic-resistant bacteria (>89%) to quinolone, beta-lactam/beta-lactamase inhibitor, cephalosporin and carbapenem antibiotics^24, 25^. Several studies have also indicated that colistin is the only remaining therapeutic option for MDRAB infections, with the percentage of resistant strains at less than 7.9%^22, 24, 25^. However, *A. baumannii* is beginning to develop resistance to colistin in settings worldwide^25, 26^.

Our study demonstrated that colistin is still effective for the treatment of *A. baumannii*. All of the *A. baumannii* strains were sensitive to colistin; thus, the relationship between the resistance to colistin and the tested phages was not available. Interestingly, *A. baumannii* strains that showed sensitivity or resistance to tigecycline were infected at a high rate by our phages, and significant differences were not observed. This phenomenon may be attributed to the function of the regulatory genes involved in tigecycline resistance, including genes related to amino acid/inorganic ion transport, metabolism and transcription^27^. These mechanisms are not directly related to the resistance of *A. baumannii* to phage infection, which would include a loss of receptors for phage absorption, the degradation of phage nucleic acids or the blocking of the phage infection process.

Nearly 50% of the antibiotic-sensitive strains of clinically isolated *A. baumannii* could be infected by our isolated phages. This result indicated that most of our phages are inherently isolated from the hospital environments. Because hospitals are locations with high antibiotic usage, the *A. baumannii* strains isolated from hospitals show a tendency toward antibiotic resistance^28^. Moreover, the development of resistance to antibiotics may cause significant fitness costs for *A. baumannii*, and these fitness costs may affect their evolution of resistance to phages^19^. A study by Kitti *et al*. also demonstrated that only MDRAB can be infected by their phage cocktails containing five phages, whereas antibiotic-sensitive *A. baumannii* cannot be infected by the same phages^29^. Recently, a mechanism of bacterial resistance to phage infection was identified, and it involves integrating phage spacer sequences in clustered, regularly interspaced short palindromic repeats (CRISPRs)^30^. These repeat and spacer sequences were accompanied by four to ten CRISPR-associated (*cas*) genes to form the so called CRISPR-Cas system^31^. This mechanism, which is similar to an adaptive immune system, can protect bacteria from phage infection. Interestingly, significant inverse correlations between antibiotic resistance and the presence of CRISPR-Cas loci were also observed in *Enterococcus faecalis* ^32^ and *Klebsiella pneumoniae* ^30^. Therefore, an evolutionary trade-off for resistance to antibiotics in the form of increased phage susceptibility may be occurring in medical environments that have high antibiotic use.

Bacterial resistance to multiple drugs involves many complex mechanisms that can be divided into two categories. The first mechanism involves these bacteria accumulating multiple genes on resistance plasmids, with each gene coding for the resistance to a specific drug. The second mechanism may involve the increased expression of genes that code for multidrug efflux pumps, which can extrude different drugs^33^. For MDRAB, a sequence analysis indicated a cluster of 45 different genes that may be involved in these two mechanisms for drug resistance^34^. Considering the many complex mechanisms required for developing antibiotic resistance, *A. baumannii* would inevitably evolve antibiotic resistance, resulting in phage susceptibility.

A recent study revealed that when the multidrug-resistant species *Pseudomonas aeruginosa* was challenged with the lytic bacteriophage OMKO1, the evolution of bacterial resistance to phage absorption led to increased susceptibility to ceftazidime (group III), ciprofloxacin (group IV), tetracycline (group VII) and erythromycin (with a mechanism similar to group I and VII)^35^ because OMKO1 utilizes the outer membrane of the efflux systems in *P. aeruginosa* as a binding site. Because the efflux systems are considered the attack target, *P. aeruginosa* would be expected to develop resistance to the phage, which would cause a genetic trade-off between efflux-mediated antibiotic resistance and phage resistance^35^. Our results are consistent with this finding because among the phage-resistant *A. baumannii* strains, most were antibiotic sensitive (groups I, III, IV and VII; Table 1). Although we observed similar results in the field environment, we speculate that the evolutionary trade-off in the bacterial resistance mechanisms was reversed^35^. The hospital environment may cause *A. baumannii* to be exposed to different groups of antibiotics, which then reduces the level of phage immunity by imposing a fitness cost on the bacteria or decreasing CRISPR-Cas activity^30^.

Phage that can infect bacteria have two abilities. The first ability is that of phage to recognize receptors on the cell surface and inject DNA into the target cell. The second is that of phage to overcome numerous bacterial anti-phage defense mechanisms (CRISPR-Cas). *A. baumannii* resistant to certain antibiotics (chloramphenicol and erythromycin) has increased production of capsular exopolysaccharide^36^. A previous study demonstrated that capsular exopolysaccharide could be the primary receptor for *A. baumannii* phage^37^. The increased number of phage binding receptors in antibiotic-resistant *A. baumannii* could cause these bacteria to suffer from a high risk of phage infection.

Recently, the tail fiber protein of ϕAB6 used in this study (Table 3) was characterized^37^, and its receptor-binding protein has polysaccharide depolymerase activity^38^. This activity can hydrolyze *A. baumannii*-specific exopolysaccharides and determine host specificity. The differences in the tail fiber proteins between ϕAB1 and ϕAB6 cause their different host ranges, despite these two phages sharing a high degree of gene conservation^38^. Replacement of the tail fiber gene has proven to alter the host specificity of ϕAB1^38^. Consequently, if the challenge from antibiotic adoption can alter the exopolysaccharide of *A. baumannii* cells^36^, specific phages may not recognize their host, resulting in different phage typing outcomes. Lai *et al*.’s study is the first report to demonstrate the interactions between specific phages and *A. baumannii* ^38^; however, this interaction cannot be the only mechanism, and the detailed recognition mechanisms may change among phages^39^. This inference can be confirmed by our results in Fig. 2 as many clinical *A. baumannii* can still be infected by both ϕAB1 and ϕAB6 (group B and D). Other phage-host recognition mechanisms are still poorly understood.

**Table 3:** The 24 specific phages used in this study to evaluate the fitness costs associated with resistance to isolated phages and antibiotics.

Podoviridae	Isolation Location	Related Reference^#^
ϕAB1	hospital sewage, Hualien	8, 14, 15, 36
ϕAB2	hospital sewage, Hualien	8, 13, 15, 36
ϕAB4	hospital sewage, Hualien	8
ϕAB5	hospital sewage, Hualien	8
ϕAB6	hospital sewage, Hualien	8, 15, 36, 37
ϕAB7	hospital sewage, Hualien	8, 15
ϕAB9	hospital sewage, Hualien	8
ϕA1	hospital sewage, Taipei	—
ϕA2	hospital sewage, Taipei	—
ϕB	hospital sewage, Taipei	—
ϕ204	city sewage, Taichung	—
ϕ385	city sewage, Taichung	—
ϕ4C08	city sewage, Pingtung	15
ϕ5C01	poultry sewage, Hualien	—
ϕ5C03	poultry sewage, Hualien	—
ϕ6C01	park pond, Hualien	—
ϕ7C01	lotus farm, Hualien	—
ϕ8C07	estuary water, Hualien	15
**Myoviridae**		
ϕ717	hospital sewage, Beijing	—
ϕAB8	hospital sewage, Hualien	—
ϕAB11	hospital sewage, Hualien	8, 15
ϕBS46	sewage, Birmingham	Laval Univ.
ϕ4C01	city sewage, Pingtung	—
**Siphovirdae**		
ϕ5C05	poultry sewage, Hualien	15

^#^Relevant reference for each specific phage.

**Figure 3:**
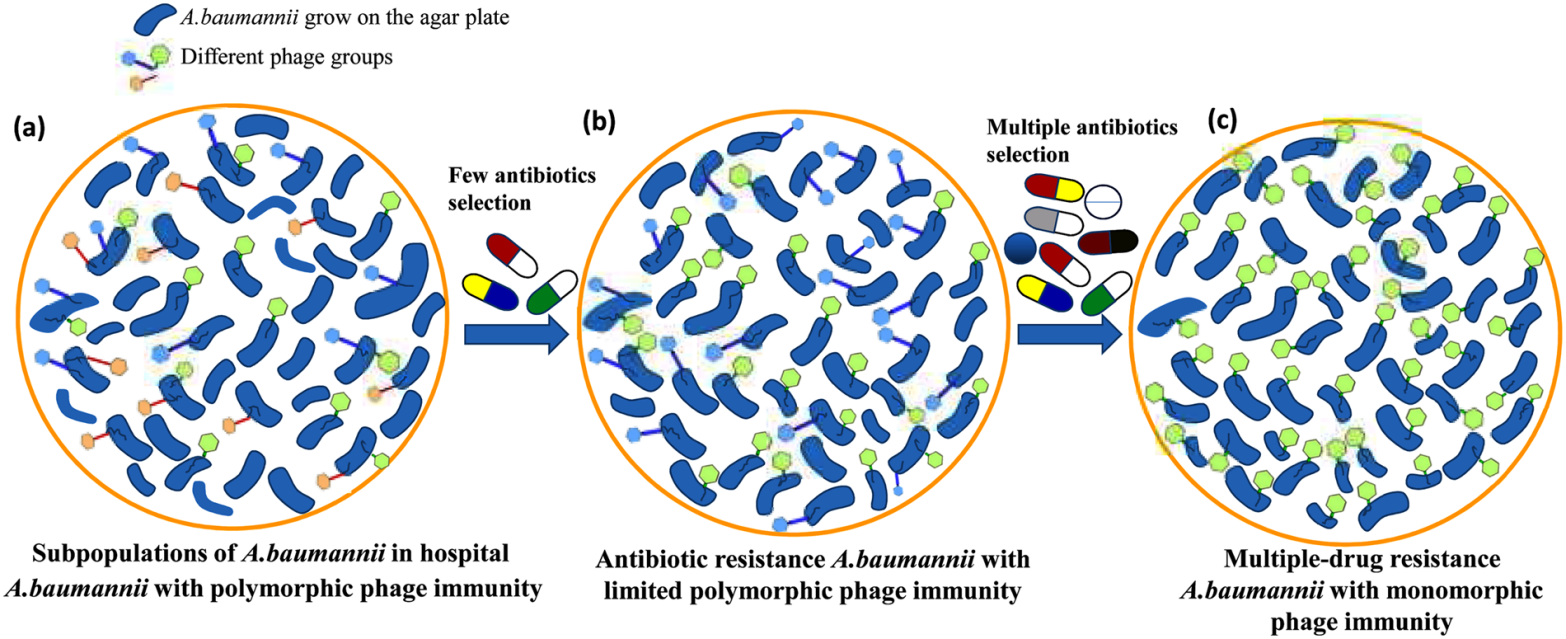
Subpopulations of the clinically isolated *A. baumannii* strains with different phage immunities: (**a**) strains showing sensitivity to antibiotics demonstrated moderate phage immunity, (**b**) strains showing resistance to a limited number of antibiotics (less than 4) demonstrated moderate phage immunity and (c) stains showing resistance to multiple antibiotics (at least 4) demonstrated low phage immunity.

In addition to the evolutionary trade-off mechanisms of bacterial resistance, we also hypothesize that when bacteria began to develop resistance to antibiotics, their phage immunity changes from polymorphic to monomorphic (Fig. 3). In the hospital environment, most *A. baumannii* can be infected by different groups of phages (Fig. 3(a)). Since these *A. baumannii* were challenged with antibiotics, the antibiotic-resistant *A. baumannii* that remained in the hospital could be more easily infected by phages because of the trade-off mechanisms. However, their phage immunity may be limited to polymorphic or monomorphic immunity (Fig. 3(b) and (c)); the phage resistance profile would be simple. This hypothesis may be validated by the characterization of our *A. baumannii* clinical strains using random amplified polymorphic DNA polymerase chain reaction (RAPD-PCR; Supplementary Information). Twenty-five strains of *A. baumannii* that were not drug resistant and that could not be lysed by any of the phages in our study were randomly chosen for analysis. The RAPD-PCR results showed that these 25 strains can be grouped into at least 10 different types according to their patterns (Supplementary Figure S1). Consequently, we suppose the source of the study strains is diverse. By contrast, *A. baumannii* strains resistant to all antibiotics in this study except for colistin and tigecycline displayed opposite results; they presented nearly the same RAPD-PCR pattern when their antibiotic resistance profiles were similar. In addition, the RAPD-PCR findings were consistent with the phage typing results. Groups A and F indicated in Fig. 2 were not analyzed by RAPD-PCR because limited strains were resistant to all antibiotics. However, the RAPD-PCR results confirmed that these strains in groups B, C, D and E indeed belong to identical clonal lineages within the groups but that clonal lineages differed among groups (Fig. 2; Supplementary Figures S2–S5). Even when these clinical strains were resistant to multiple drugs but could not be lysed by any of the phages, their fingerprints were very similar (Supplementary Figure S6). Given these results, we hypothesize that drug-resistant bacteria might have a natural selection advantage due to gene consistency. Conversely, non-drug resistant bacteria that do not experience selection show a variety of results.

Consequently, the higher phage susceptibility of the clinical *A. baumannii* strains may be caused by the selective pressure of adopting resistance to multiple antibiotics. After removing the antibiotic-sensitive strains, the multiple drug-resistant *A. baumannii* strains demonstrated more homogeneous gene content, and their phage immunity could be limited to polymorphic or monomorphic immunity. Although we have done as much as possible to isolate more phages, these 24 phages are likely only a small part of the environment. Since the phage immunity of *A. baumannii* was defined by our limited phages, the hospital-wide *A. baumannii* resistance to fewer phages, the bacteria would have a higher likelihood of being infected by our limited phages.

In our study, the phage IP% for antibiotic-resistant *A. baumannii* was 84%, which indicates that phage therapy is a potential approach for treating MDRAB infections. The advantage of phage therapy is that it overcomes the limitations of antibiotics in killing antibiotic-resistant bacteria, and it can also restore the effectiveness of antibiotics that are considered less therapeutically valuable. Under phage selective pressure, bacteria may develop decreased susceptibility to antibiotics, thus allowing historically effective antibiotics to be applied for the treatment of infection^35^. However, phage therapy also faces a similar dilemma to antibiotic use in that bacteria may develop resistance to phages^18^, although the phages may also adapt to that resistance and regain the ability to infect bacteria^18, 19^. This coevolutionary arms race between phages and bacteria involves many complex mechanisms related to defense and counter defense^40^. Because bacteria can develop resistance to both antibiotics and phages, several researchers have suggested the combined use of antibiotics and phages^41–43^. The selective pressure from antibiotics could be valuable in making the phage immunity of *A. baumannii* more monomorphic. This process could help phage therapy kill all antibiotic-resistant *A. baumannii* more easily and completely with limited polymorphic phage immunity. In addition, such combined therapies may be useful for minimizing the possibility of resistance evolution to phages and antibiotics because simultaneous multiple mutations for both antibiotics and phage resistance are relatively rare^42, 43^.

Several microbial typing methods have been developed for clinical or epidemiological purposes. Among these methods, phage typing was developed in 1980 for typing *Acinetobacter* species, and it has been recommended as a good typing system for hospital outbreaks^44, 45^. Among the clinically isolated *A. baumannii* in our study (403 isolates), 305 were typeable (75.6%), and this typeable percentage was nearly equivalent to that in the study by Joly-Guillou *et al*. (75%)^44^, but it was much higher than that observed in the study by Bouvet *et al*. (41%)^45^. In addition to identifying the bacterial strain, our study also demonstrated that the antibiotic-resistant profiles of clinically isolated *A. baumannii* could be predicted by the phage typing results. The observation of similar phage typing results among *A. baumannii* isolates indicates that these species may have similar antibiotic-resistance profiles. A previous study also indicated that phage typing could be used to identify *A. baumannii* strains resistant to beta-lactams, aminoglycosides or fluoro-quinolone^44^.

Although phage typing should be extended to other phages to increase the bacterial typeable percentage, the main advantage of phage typing for the identification of bacterial strains and antibiotic-resistance profiles is the time savings. At least 48 h are required to identify bacteria and ascertain their drug resistance profiles using commercially available machines, such as the Phoenix instrument, whereas the detection time may be reduced by 24 h using phage typing, depending upon the implemented method. In low-income areas or in certain emergency situations, phage typing may be a potential method for determining the correct therapeutic treatment within a limited time. However, we still recommend the use of phage typing in parallel with other methods, such as biotyping or protein typing, to provide more suitable information. Further studies should be performed to identify additional phages to increase the typeable strains and improve the application of phages in phage cocktails.

## Methods

### Isolation and spot test of the bacteriophages

This study was conducted in the main tertiary referral medical center serving eastern Taiwan. Clinical *A. baumannii* isolates were collected from January 2014 to March 2015. In the past 3 years, we have continuously and successfully identified 24 active phage isolates against *A. baumannii* from sewage, park ponds, lotus farms, or river water by the phage research team in our hospital. Our study is located in an eastern city, Hualien; however, we also collected phages from different cities, including Taipei, Taichung, and Pingtung. These cities represent different locations in the northern, western, and southern regions of Taiwan, respectively. The distance from Hualien to these cities is at least 200 km. In addition, phages were also collected from Beijing and Birmingham in this study. Among these phages, ϕBS46 was provided by Laval University, and it was also isolated from sewage. Several of these phages have been subjected to genomic analyses^12, 14^ and endolysin applications^13^, and methods of applying these phages for environmental cleaning have been evaluated^15, 16^. Specific characteristics of the 24 phages used in this study are shown in Table 3.

### Host range analysis

A host range analysis of the 24 bacteriophages was performed via spot tests on all of the isolated *A. baumannii* strains. One hundred microliters (1.0 × 10^7^ cells) of fresh bacterial culture (OD_600_ = 1.0) was added to 10 ml of soft LB agar (0.7%), mixed gently, and then poured onto a regular LB agar plate. Subsequently, 2-µl aliquots of the phage suspension (2.0 × 10^8^ PFU) were spotted on the lawn of the bacteria. The plates were dried in a biosafety cabinet for 10 min and incubated at 37 °C for 18–20 h. The clearance zone indicating lysis at the spot of phage inoculation implied that the host was sensitive to the phage.

### *A. baumannii* isolates and identification

The chromogenic agar CHROMagar Acinetobacter (CHROMagar Microbiology, Paris, France) was applied to rapidly identify the *Acinetobacter* strains. Subsequently, the *A. baumannii* strains recovered on the selective chromogenic agar were identified by a Vitek system (bioMérieux, Marcy-l’Etoile, France). A previously described multiplex PCR-based assay was performed to identify the isolates at the species level^46^. Three pairs of primers targeting the *recA* and *gyrB* genes and the 16S–23S rRNA intergenic spacer region could differentiate *A. baumannii*, *A. nosocomialis* and *A. pittii* ^46^. The resistance of *A. baumannii* to different antibiotics was confirmed using the broth-dilution method in accordance with the 2012 Clinical and Laboratory Standards Institute guidelines.

### Resistance of *A. baumannii* to phages and eight antibiotic categories

To test the resistance of *A. baumannii* to the isolated phages, we sequentially applied different single phages for phage typing. The agar overlay method was applied, and the resistance of *A. baumannii* to the phages was based on whether these phages were capable of forming plaques^47^. If a single phage was capable of forming plaques on the *A. baumannii* cells, then the *A. baumannii* strain was defined as phage sensitive. However, if none of the phages were capable of forming plaques on the *A. baumannii* cells, then the *A. baumannii* strain was referred to as phage resistant.

In addition to determining the resistance of *A. baumannii* to different phages, we also determined the resistance of the clinically isolated strains to antibiotics. In this study, the antibiotic resistance of *A. baumannii* was divided into eight categories according to Fair and Tor’s review article^48^: (I) Aminoglycoside (amikacin and gentamicin), (II) Beta-lactam/beta-lactamase inhibitor combination (tazobactam-piperacillin), (III) Cephalosporin (ceftazidime and cefepime), (IV) Quinolone (ciprofloxacin and levofloxacin), (V) Polymyxin (colistin), (VI) Sulfonamides (baktar), (VII) Tetracycline (tigecycline) and (VIII) Carbapenem (doripenem, imipenem and meropenem). The resistance of *A. baumannii* to the different antibiotic categories was confirmed using the broth-dilution method in accordance with the 2012 Clinical and Laboratory Standards Institute guidelines. All of the *A. baumannii* strains (N = 403) were subjected to a carbapenem resistance test. However, the resistance of *A. baumannii* to the other groups of antibiotics was performed based on the clinical treatment requirements; thus, certain strains were not subjected to all of the antibiotic susceptibility tests.

### Statistical analysis

We used the Kolmogorov-Smirnov test to determine whether the sample data were normally distributed. After the statistical analysis, the data were analyzed with parametric tests because the probability associated with the Kolmogorov-Smirnov test for normality was >0.05. We used the chi-square test or Fisher’s exact test to examine differences in the distribution of *A. baumannii* susceptibility to the tested antibiotics and phages. In addition, the odds ratio (OR) was obtained from a binary logistic regression model between the outcome variable, which was ‘phage infected,’ and the independent variables, which were the various numbers of antibiotics to which *A. baumannii* was resistant. Finally, the relationship between the phage infection profiles and the normalized drug resistant percentages for the eight groups of antibiotics were also evaluated using a chi-squared test.

## Electronic supplementary material

Supplementary Information
