# Supplementary material for: Clinical Antibiotic-resistant Acinetobacter baumannii Strains with Higher Susceptibility to Environmental Phages than Antibiotic-sensitive Strains
Source: Sci Rep. 2017 Jul 24;7:6319. doi: 10.1038/s41598-017-06688-w (PMC5524697; doi:10.1038/s41598-017-06688-w)
Supplement: Supplementary file 1 — Supplementary Information [file 41598_2017_6688_MOESM1_ESM.pdf]

Clinical Antibiotic-resistant *Acinetobacter baumannii* Strains with Higher Susceptibility to Environmental Phages than Antibiotic-sensitive Strains

Li-Kuang Chen[12], Shu-Chen Kuo[3], Kai-Chih Chang[4], Chieh-Chen Cheng[5], Pei-Ying Yu[5], Chih-Hui Chang[5], Tren-Yi Chen[6], and Chun-Chieh Tseng[5*]

Supplementary Information

Characterization of clinical *A. baumannii* by RAPD-PCR

Random amplified polymorphic DNA polymerase chain reaction (RAPD-PCR) was performed with M13 universal primers. A loopful of bacterial growth containing 2 to 3 small representative colonies was suspended in 100 μL of sterile distilled water in a 1.5 ml Eppendorf. These were heated for 10 min at 95℃, cooled on ice, and centrifuged at 12,000×g for 20 seconds to remove all cell debris. These crude DNA extracts were either frozen at 20℃ or were kept on ice for immediate use, and 2 μL of these extracts were used in the 20 μL PCR mixtures without any further purification.

For PCR, 13.5 μL of sterile distilled water, 1 μL of each primer (10μM), 1 μL of 2.5 mM dNTP's, 2 μL of 10X PCR buffer and 0.5 μL of Taq polymerase was used. M13 primers were obtained from Protech Technology. The primer sequences were 5'-GTA AAA CGA CGG CCA GTG AA- 3' (forward primer) and 5'-GGA AAC AGC TAT GAC CAT GA- 3' (reverse primer). The PCR conditions were as follows: 94℃ for 2 min, followed by 35 cycles of 94℃ for 30 seconds, 45℃ for 1 min, and 72℃ for 40 seconds, with a final extension at 72℃ for 5 min. The PCR end products from all of the isolates were analyzed by electrophoresis on 2% agarose gels (100 mV, 40 min). The gels were stained with EtBr for 5 min and examined on a UV transilluminator.

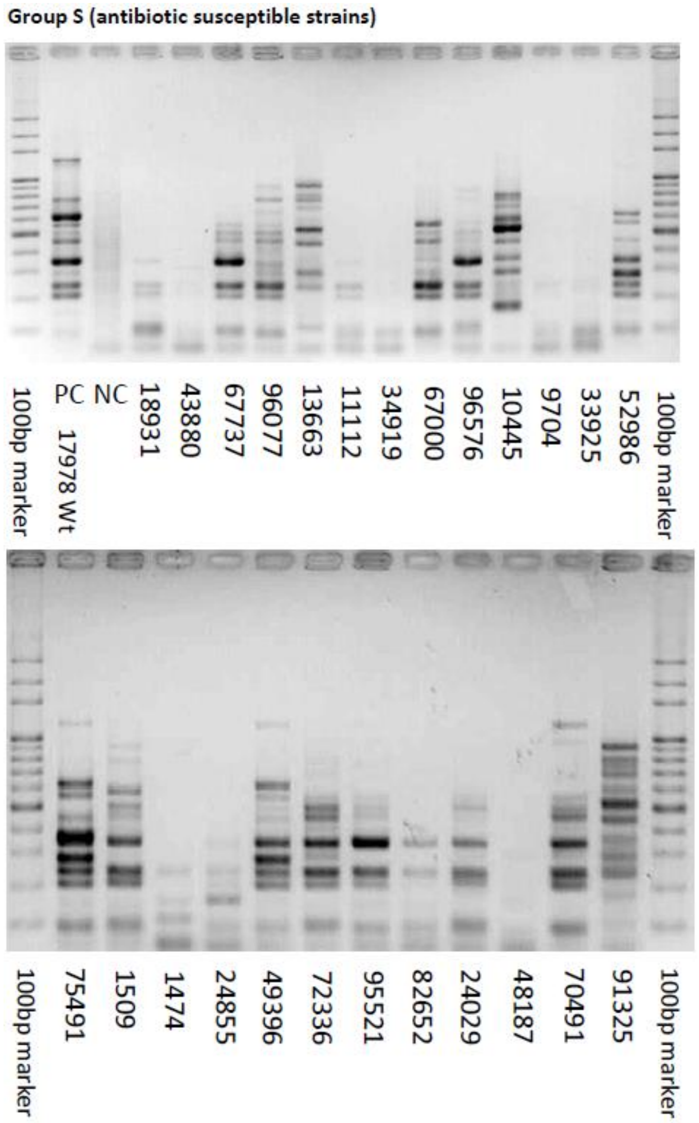

Figure S1. RADP-PCR fingerprinting of representative clinical strains of *A. baumannii* with the M13 primers. These 25 strains of *A. baumannii* are not drug-resistant and cannot be lysed by any of the phages in our study. The RADP-PCR results showed that strains 67737, 96576, 1509, 72336, 95521, 24029, and 70491 seem to be demonstrate the same pattern. Strains 13663 and 91325 can be separated into a second group. Strains 75491 and 49396, which have similar bands, can be grouped by another pattern. The other strains belong to different types. These 25 strains can be grouped into at least 10 different types according to their patterns. PC: positive control; *A. baumannii* (ATCC 17978); and NC: negative control.

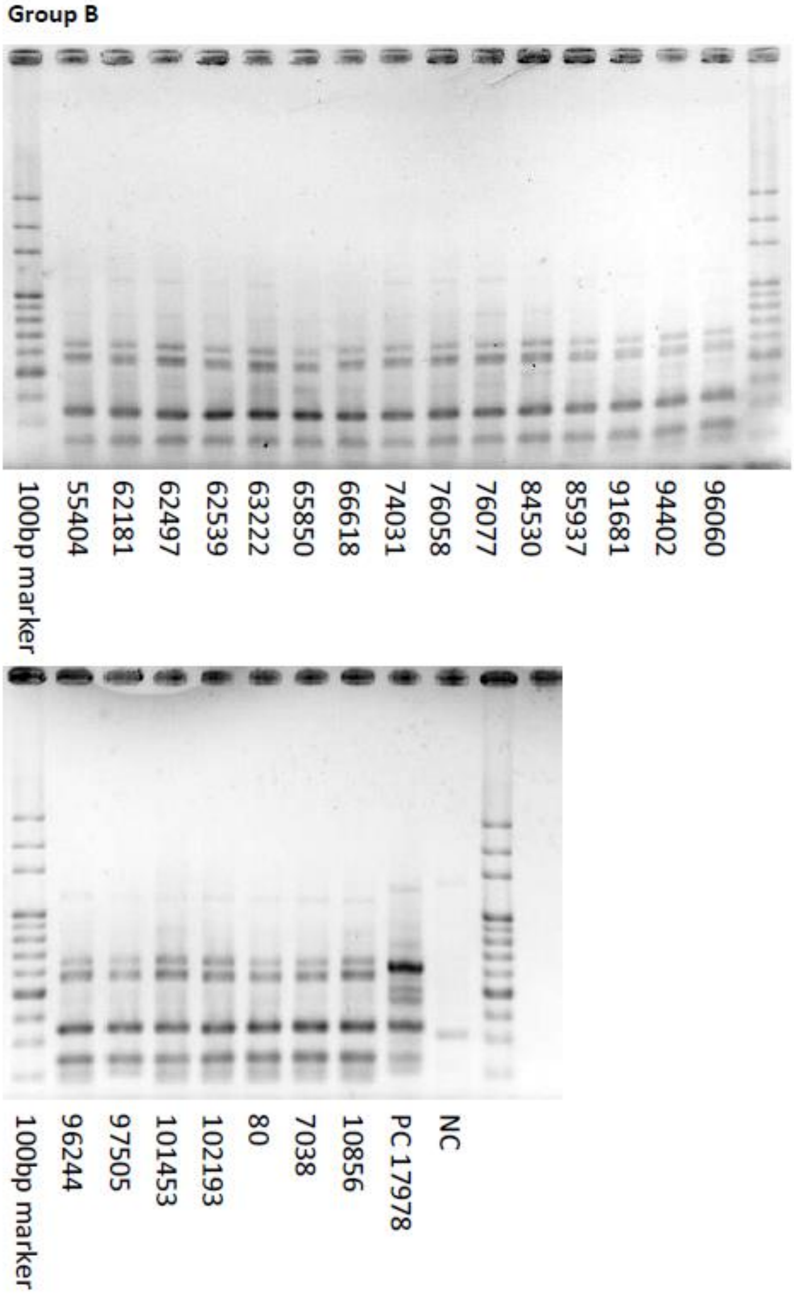

Figure S2. RADP-PCR fingerprinting of representative clinical strains of *A. baumannii* (group B) with the M13 primers. Group B (defined in Table 4) contains 22 *A. baumannii* strains that show multiple drug resistance and can by identified by different phage typing. These 22 strains showed the same RAPD-PCR pattern. PC: positive control, *A. baumannii* (ATCC 17978), and NC: negative control. The definition of multiple-drug resistance is that *A. baumannii* is resistant to all antibiotics in this study except for colistin and tigecycline.

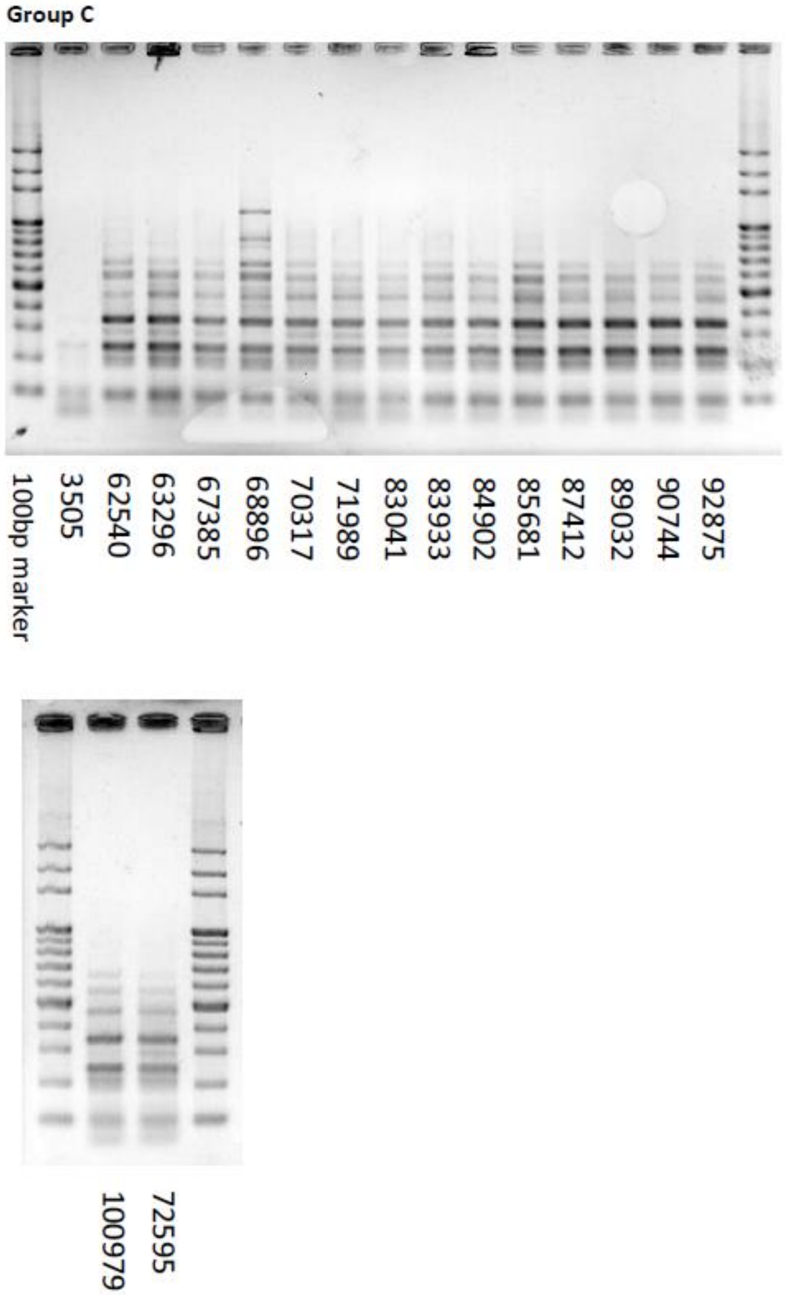

Figure S3. RADP-PCR fingerprinting of representative clinical strains of *A. baumannii* (group C) with the M13 primers. Group C (defined in Table 4) contains 17 strains of *A. baumannii* that shows multiple drug resistance and can be identified by bacteriophage typing. Nearly all of the strains can be identified as having the same pattern, except for strain 68896. These 17 strains can be grouped into 2 different types according to their patterns. The definition of multiple-drug resistance is that *A. baumannii* is resistant to all of the antibiotics in this study except for colistin and tigecycline.

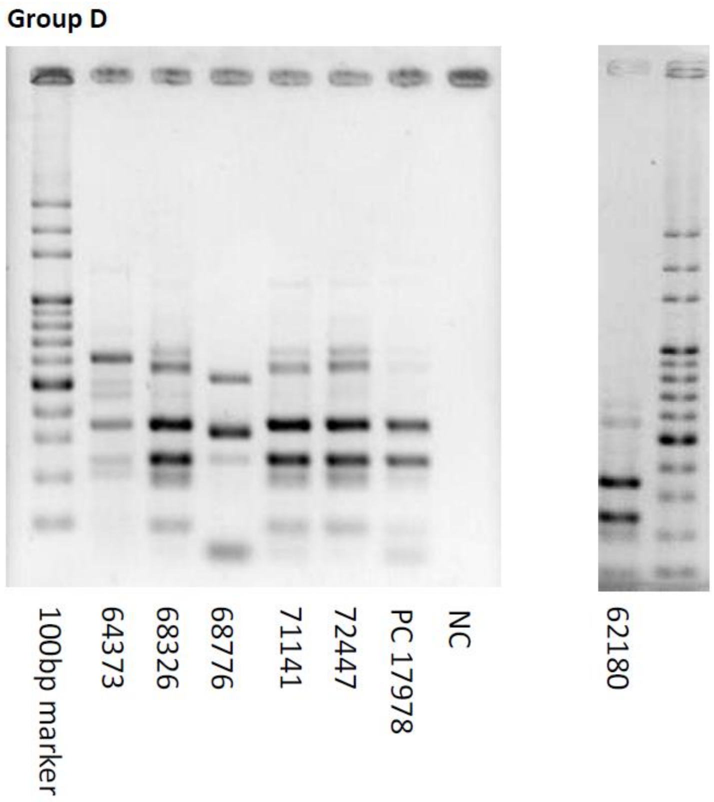

Figure S4. RADP-PCR fingerprinting of representative clinical strains of *A. baumannii* (group D) with the M13 primers. Group D (defined in Table 4) contains 6 *A. baumannii* strains that show multiple drug resistance and can be identified by bacteriophage typing. Strains 64373, 68326, 71141, 72447, and 62180 belong to the same pattern. Strain 68776 belongs to the other pattern. These 6 strains can be grouped into 2 different types according to their pattern. PC: positive control, *A. baumannii* (ATCC 17978), and NC: negative control. The definition of multiple-drug resistance is that *A. baumannii* is resistant to all of the antibiotics in this study except for colistin and tigecycline.

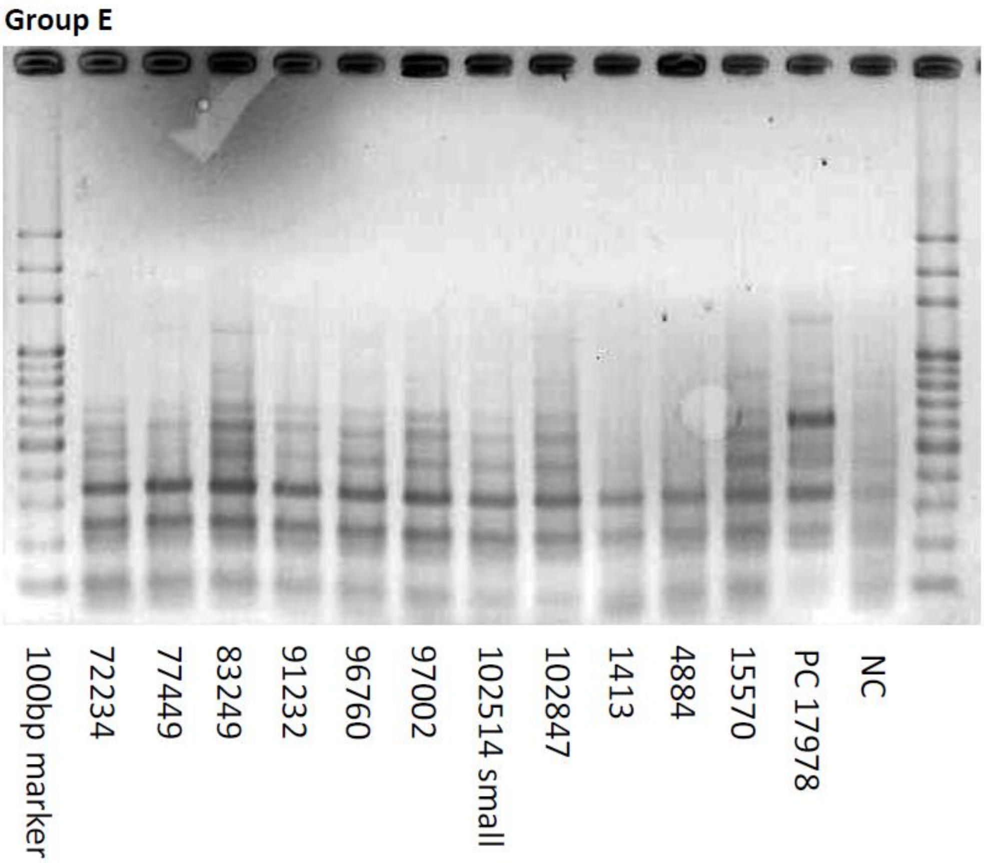

Figure S5. RADP-PCR fingerprinting of representative clinical strains of *A. baumannii* with M13 primers. Group E (defined in Table 4) contains 11 strains of *A. baumannii* that show multiple drug resistance and can be identified by bacteriophage typing. These 11 strains also showed a similar RAPD-PCR pattern. PC: positive control, *A. baumannii* (ATCC 17978), and NC: negative control. The definition of multiple-drug resistance is that *A. baumannii* is resistant to all of the antibiotics in this study except for colistin and tigecycline.

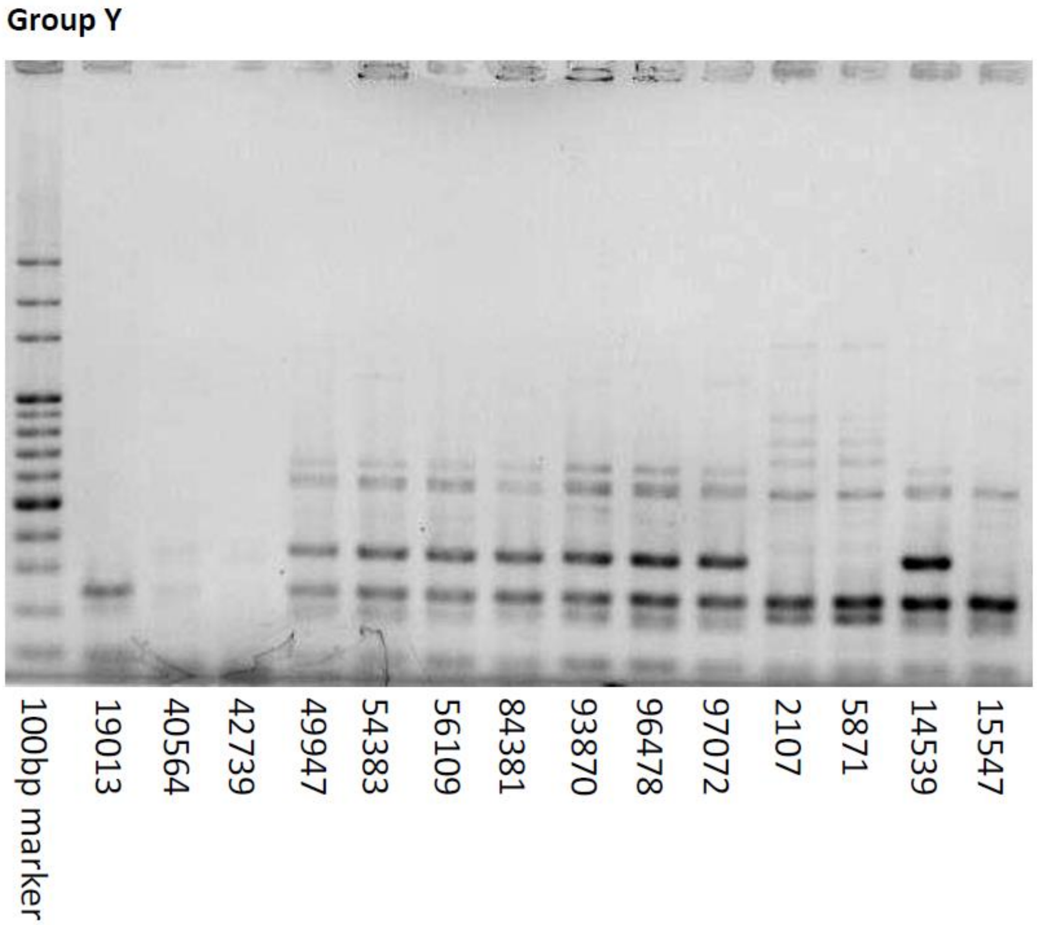

Figure S6. RADP-PCR fingerprinting of representative clinical strains of *A. baumannii* with the M13 primers. Group Y contains 14 strains of *A. baumannii* that show multiple drug resistance and cannot identify by phage typing. Strains 49947, 54383, 56109, 84381, 93870, 96478, 97072, and 14539 belong to the same pattern. Strains 2107, 5871, and 15547 can be classified into another pattern due to the lack of a band between 300 bp to 400 bp. No bands were observed for strains 19013, 40564, and 42739. These 14 strains can be grouped into 3 different types according to their pattern. The definition of multiple-drug resistance is that *A. baumannii* is resistant to all of the antibiotics in this study except for colistin and tigecycline.
